# Loss of Recurrent Laryngeal Papillomatosis following Postsurgical Treatment with dsRNA Interferon Inducer

**DOI:** 10.1155/2024/6044987

**Published:** 2024-07-08

**Authors:** Alina Sultanova, Liba Sokolovska, Maksims Cistjakovs, Andrejs Lifsics

**Affiliations:** ^1^ Institute of Microbiology and Virology Riga Stradiņš University, Rātsupītes 5 LV-1067, Riga, Latvia; ^2^ Department of Otorhinolaryngology Riga Stradiņš University, Pilsoņu 13 LV-1002, Riga, Latvia

## Abstract

Laryngeal papillomatosis (LP) is the most common benign laryngeal tumor in children, but it can affect both children and adults. Although benign, this condition still remains hard to treat and negatively affects patient quality of life as it can spread to the adjacent respiratory tract, recurs, and requires repeated medical intervention. As the surgical removal of papillomas with the preservation of normal mucosa is the only standard of care, there is still no standard for adjuvant therapy. In this case report, we describe the course of recurrent laryngeal papillomatosis in a Caucasian male from 2009 to 2016 and the positive response to dsRNA-based antiviral drug treatment in the complete resolvement of the condition.

## 1. Introduction

Laryngeal papillomatosis (LP) (also referred to as respiratory recurrent papillomatosis) is the most common benign laryngeal tumor in children, but it can affect both children and adults. The LP incidence is estimated to be 4.3 per 100,000 in children and 1.8 per 100,000 in adults. Although benign, this condition still remains hard to treat and negatively affects patient's quality of life as it can spread to the adjacent respiratory tract, recurs, and requires repeated medical intervention [1, 2].

This condition is characterized by the overproliferation of benign squamous papillomas and has a viral etiology. Most notably, human papillomaviruses (HPVs) are cited as the main viral agents associated with the disease [3], with the two low-risk HPVs, HPV6 and HPV11 most commonly involved in LP. HPV11 in particular, has been associated with a more severe disease [4–6].

At the moment, there is no “cure” for LP. Surgical removal of papillomas with the preservation of normal mucosa is the standard of care [7, 8]. Adjuvant therapy can be implemented only if there are more than four surgical procedures in a year, rapid papilloma regrowth, airway compromise, and/or distal multisite spread of the disease. Options for adjuvant therapy include cidofovir, photodynamic treatment, bevacizumab, interferon, indol-3 carbinol, and proton-pump inhibitors. Adjuvant therapies are often off-label and can have a wide range of responses to treatment. Peginterferon alpha-2a was the most commonly used interferon, but it has been replaced by cidofovir due to its high side effects and the emergence of cidofovir [7, 9].

Therapeutic vaccination against HPV has also been proposed as a treatment option for LP. Although not many large studies have investigated the effects, a recent meta-analysis revealed that vaccination significantly reduced the number of surgical procedures per month and increased the intersurgical period [10].

In addition, we believe that there are several other antiviral medications on the market from which LP patients could benefit greatly. One of these medications was used in the treatment of the described case. It contains bacteriophage-derived dsRNA and is a potent inducer of endogenous type I interferons (IFNs) and acts as a polyfunctional and wide-spectrum antiviral drug [11]. It has been shown to induce and activate several enzymes of the IFN system involved in the blockade of translation of infected cells [12–14]. Furthermore, it is an affordable and over-the-counter medication.

In this case report, we describe the course and the complete resolvement of recurrent laryngeal papillomatosis in a Caucasian male who had undergone 14 surgeries between 2009 and 2016. The purpose of this case report is to highlight an affordable, over-the-counter dsRNA-based antiviral drug as a potential candidate for LP adjuvant therapy.

## 2. Case Presentation

At age 28 (year 2009), a Caucasian male sought medical attention due to experiencing difficulty in speaking and significant changes in the timbre of his voice. The patient was a sailor by occupation and spent up to 6 months on the voyage. The patient was a nonsmoker and had no underlying medical conditions (HIV-negative and HCV-negative).

A detailed examination of the patient revealed laryngeal papillomatosis. It was decided to remove the formation with the help of a CO_2_ laser under general anesthesia. During CO_2_ laser therapy, strengthened double bag tracheal intubation and low oxygen (<30%) lung ventilation were required. Visible papilloma lesions were removed with a CO_2_ laser after pedestal laryngoscopy and laryngeal endoscopy. Following histological examination, postsurgical material showed no evidence of malignancy.

Since the first surgery, the patient had undergone an additional 13 surgeries (in the period from 2009 to 2016) with a mean interval of 6.45 ± 2.29 months between surgeries due to the recurrence of the lesion. The shortest interval between surgeries was four, and the longest was ten months. In the postsurgical period, the patient was prescribed five days of a home regime during the first two weeks to significantly decrease verbal communication (speak quietly if necessary). For pain relief, ibuprofen was prescribed 400 mg per oral (max 3x per day) and the patient was followed up after one month. The patient was prescribed general strengthening biological additives, such as vitamins and homeopathic treatment with immunostimulatory properties during the whole period. The patient led an active lifestyle.

Only after the 14th surgery, the postsurgical material was sent for the first HPV testing. At this time, the patient was 34 years old. In addition, blood analysis was ordered to assess the patient's immunological status.

The postsurgical specimen was received as tissue debris (multiple small pieces of around 2 mm) in phosphate-buffered saline (PBS). One piece of tissue was used for DNA extraction using the phenol-chloroform method. The concentration and the quality of the extracted DNA were measured spectrophotometrically (NanoDrop ND-1000 Spectrophotometer, Thermo Fisher Scientific, Waltham, MA, USA). 200 ng of the extracted DNA was taken for polymerase chain reaction (PCR).

**Figure 1:**
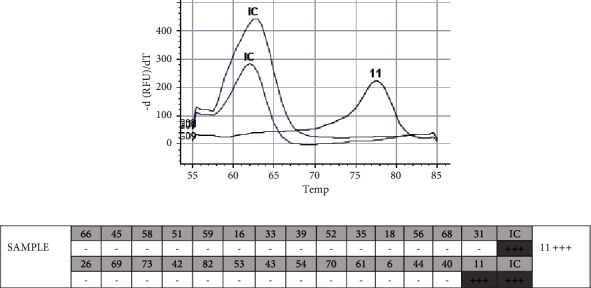
Detection and genotyping of HPV using Anyplex™ II HPV28 kit (Seegene, South Korea). HPV types (divided into two groups) detection is made in two parallel reactions. Internal control (IC) is included for each detection group. (a) Representation of PCR reaction results in the form of melt peaks. (b) Representation of PCR reaction results in the form of a table reflecting all HPV types genotyping included in this test. The minus symbol (−) represents a negative result. The plus symbol (+) represents a positive result (one to three pluses represent a semiquantitative result).

HPV testing was performed using the Anyplex™ II HPV28 PCR kit (Seegene, South Korea), which allows genotype 28 of the most important low- and high-risk HPV types and provides semiquantitative results of the amount of HPV detected in the sample. PCR testing revealed that the postsurgical material harbored only one HPV genotype, HPV11. In addition, the test revealed a high viral load (+++) of HPV11 (Figure 1).

Blood analysis revealed changes in lymphocyte subpopulations (Table 1, 14.01.2016). CD3 and CD4 relative cell counts were decreased to 60 (66–80) and 27 (35–50), while CD16 was elevated to 30 (8–17). The CD4/CD8 ratio was decreased as well to 0.8 (1.3–2.3). The lymphocyte changes observed, especially the decrease in CD3, CD4, and CD4/CD8, could be indicative of an immunosuppressed state of the patient. Levels of IgG, IgM, and IgA antibodies were within normal values. In addition, serum carcinoembryonic antigen (CEA) and antinuclear antibodies (ANA) were analyzed to confirm the absence of malignancy and autoimmune disease.

At this time, the patient was prescribed a drug based on lyophilized dsRNA (orally as a spray of 0.1 mg × 3 per day for three weeks), that is, Larifan (Larifan Ltd., Riga, Latvia), which is a nationally well-known antiviral drug. As of right now, the State Agency of Medicines of the Republic of Latvia has approved and registered Larifan for use in treating herpesvirus infections and secondary immunodeficiency (Reg. No. 04-0230); however, it was shown that it could also be effective in the treatment of other infections, such as SARS-CoV-2 and papillomaviruses [14, 15]. After the course of the prescribed therapy, the symptoms no longer appeared.

After a year and a half, the patient visited the doctor for a follow-up examination. Examination did not reveal recurrent papillomatosis. Immunological parameters at the time of the visit also returned to normal (Table 1, 9.06.2017). At the insistence of the patient, three doses of the HPV vaccine Gardasil 9 (HPV6, 11, 16, 18, 31, 33, 45, 52, and 58) were also administered during this period (one month between the first and second round, and nine months after two, the third round) to further prevent relapse.

Now, seven years after the final surgery, treatment with Larifan, and HPV vaccination, the patient has had no relapse.

## 3. Discussion

At the moment, surgical interference is the golden standard for treating LP. However, this procedure does not save patients from the recurrence of LP, with some patients undergoing up to four surgeries in a year [7, 8]. The surgery is accompanied by total anesthesia, which negatively affects the host's immune system. It has been shown that total anesthesia can cause immunosuppression, which in turn could affect the viral activity and could aid the prolongation of HPV persistence [16, 17].

Adjuvant therapy could help reduce the number of surgeries, yet there are no clear guidelines, and the effectiveness of the drugs used varies substantially [8]. Therapeutic approaches targeting the etiological agent of LP-HPV present a new approach to disease management and the prevention of recurrence after surgery.

In this case report, we presented a case of recurrent LP in which a male patient had undergone 14 surgeries between 2009 and 2016. After the final surgery, the postsurgical material was tested for HPV, and the patient was treated with an antiviral interferon inducer based on lyophilized dsRNA. In addition, the patient was vaccinated against HPV with Gardasil 9.

After the lyophilized dsRNA treatment, the patient did not experience any disease recurrence. It is an affordable, accessible, over-the-counter medication. It is a heterogeneous mixture of dsRNAs obtained biotechnologically from *Escherichia coli* cells infected with f2sus11 amber mutant bacteriophages. dsRNA is a well-known and potent interferon inducer, and studies have demonstrated the wide-reaching effects Larifan exerts on immune cells beyond interferon [18]. Studies have explored this drug as an antiviral and antitumor treatment option with promising results in cervical cancer, recurrent herpes simplex infection, chlamydia trachomatis infection models, and most recently, even SARS-CoV-2 [14, 19–21].

The postsurgical material of the patient was tested for HPV after the final surgery and revealed the presence of HPV11 with a high viral load. HPV11 has often been connected with a more aggressive LP and possibly, in the described case, could partly explain the number of surgeries experienced in the 5-year period and the sometimes very short intersurgical period [5]. HPV11 is one of the HPV types included in the Gardasil 9 vaccine, and even though we cannot clearly describe the effects that the vaccination with Gardasil 9 had on the described case, we believe that it played a role in the long-term maintenance of the disease-free state of the patient. Moreover, further analysis of HPV types and treatment options utilized could reveal correlations that could help predict treatment results.

The frequent surgeries compelled the patient to actively search for more effective treatments. It was the patient's initiative to try an antiviral drug and receive an HPV vaccination. Moreover, the direct communication with the patient during the report's writing helped acquire more accurate information for problem resolution. This shows that the treatment of LP should be improved to decrease the implementation of invasive surgery.

Another question should be raised for further studies, that is, if recurrent LP is caused by reinfection via relation with an infected partner or due to reactivation of HPV, then the HPV latency period is extremely variable; usually 3–6 months, but latency periods of many months or even decades have been reported [22, 23]. This period exactly coincides with our case; however, repeated contact with the infected partner and reinfection could not be excluded, but due to the specificity of the patient's occupation (sailor) and the fact that he has divorced since the first surgery, it is very hard to track.

The presented case highlights the problematic nature of LP, its ability to recur, the necessity for repeated and frequent surgical interventions, and the psychological toll this disease can have on an otherwise healthy young individual. The observations gathered in this case report shed light on a new adjuvant therapy candidate, lyophilized dsRNA, which could benefit patients suffering from recurrent LP due to its antiviral and immunomodulatory effects.

## 4. Conclusions

After a long history of recurrent laryngeal papillomatosis, the patient was finally cured and the antiviral drug based on lyophilized dsRNA seemed to play an important role in the treatment. In addition, HPV vaccination should be considered as an additional method for LP prophylaxis; however, more in-depth studies are required to make strong recommendations.

## Figures and Tables

**Table 1:** Patient's blood cell analysis.

Date	14.01.2016, first testing		09.06.2017, after LP symptoms' disappearance		Reference range^*∗*^
Blood cells' subpopulations	%	Absolute	%	Absolute	
Lymphocytes		1800		2270	
CD3	60	1050	70	1600	66–80
CD4	27	486	39	880	35–50
CD8	32	576	29	680	23-38
CD4/CD8		0.8		1.58	1.3–2.3
CD19	8	144	8	190	9–18
CD16	30	540	19	460	8–17
Other analytes					
IgG antibodies	14.3				7.0–16.0
IgA antibodies	2.94				0.7–4.00
IgM antibodies	0.8				0.4–2.3 g/l
Carcinoembryonic antigen (CEA)	<0.50				<0.50 ng/ml
Antinuclear antibodies (ANA)	Negative				Negative

^*∗*^Reference provided by Pauls Stradins Clinical University Hospital's Department of Immunology.

## Data Availability

All relevant data are available upon request from the corresponding author.
